# Direct Observation of the Layer-by-Layer Growth of ZnO Nanopillar by *In situ* High Resolution Transmission Electron Microscopy

**DOI:** 10.1038/srep40911

**Published:** 2017-01-18

**Authors:** Xing Li, Shaobo Cheng, Shiqing Deng, Xianlong Wei, Jing Zhu, Qing Chen

**Affiliations:** 1 Key Laboratory for the Physics and Chemistry of Nanodevices and Department of Electronics, Peking University, Beijing 100871, P.R. China; 2 National Center for Electron Microscopy in Beijing, School of Materials Science and Engineering, The State Key Laboratory of New Ceramics and Fine Processing, Key Laboratory of Advanced Materials (MOE), Tsinghua University, Beijing 100084, P.R. China; 3 Center for Nano and Micro Mechanics, Tsinghua University, Beijing 100084, P.R. China

## Abstract

Catalyst-free methods are important for the fabrication of pure nanowires (NWs). However, the growth mechanism remains elusive due to the lack of crucial information on the growth dynamics at atomic level. Here, the noncatalytic growth process of ZnO NWs is studied through *in situ* high resolution transmission electron microscopy. We observe the layer-by-layer growth of ZnO nanopillars along the polar [0001] direction under electron beam irradiation, while no growth is observed along the radial directions, indicating an anisotropic growth mechanism. The source atoms are mainly from the electron beam induced damage of the sample and the growth is assisted by subsequent absorption and then diffusion of atoms along the side 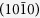 surface to the top (0002) surface. The different binding energy on different ZnO surface is the main origin for the anisotropic growth. Additionally, the coalescence of ZnO nanocrystals related to the nucleation stage is uncovered to realize through the rotational motions and recrystallization. Our *in situ* results provide atomic-level detailed information about the dynamic growth and coalescence processes in the noncatalytic synthesis of ZnO NW and are helpful for understanding the vapor-solid mechanism of catalyst-free NW growth.

Due to their excellent mechanical, optical and electronic properties, various nanowires (NWs) have been successfully synthesized through vapor-phase transport method^1^, metalorganic vapor phase epitaxy^2^,^3^, chemical vapor deposition^4^, *etc*. Although the NWs’ geometry and growth can be easily controlled with catalyst particles, catalyst-free methods are important for the fabrication of pure NWs and for the easiness that no catalyst preparation is needed. Many kinds of NWs and a variety of more complex morphologies have been fabricated using vapor-solid (VS) method in the absence of a catalyst. The spontaneous catalyst-free NW growth can be driven by screw dislocation^5^, twin boundary and stacking fault^6^,^7^, anisotropic surface energy or assisted by oxides^8^,^9^. However, contradictory mechanisms have been reported in the catalyst-free growth of NWs due to the lack of crucial information on the growth at atomic level.

*In situ* transmission electron microscopy (TEM) is a powerful method to study the growth dynamics and kinetics at atomic scale. Through *in situ* TEM study, the growth processes of NWs with metal catalysis^10^,^11^ and two-dimensional MoS_2_ flakes^12^ have been carefully demonstrated, and the complicated physicochemical processes related to the nanomaterial growth have also been well *in situ* monitored and explained at atomic level, such as the coalescence of nanocrystals^13^,^14^, lateral atoms displacements and rotations^15^,^16^,^17^, and surface reconstructions^18^, *etc*. Recently, with *in situ* environmental TEM, the noncatalytic metal oxide NW growth through thermal oxidation has been observed with lattice resolution, and the NWs have been suggested to grow through the oscillatory mass transport process^19^ or layer-by-layer growth induced by surface nucleation at the edge of twin boundary ridges^20^. However, understanding the NW growth process in the VS mechanism dynamically at atomic level still remains a challenge.

As a representative nanomaterial, ZnO, especially wurtzite (WZ) NWs with <0001> growth direction, are promising candidates for a wide range of applications in ultraviolet optoelectronics^21^,^22^, electromechanics^23^ and gas sensors^24^,^25^. Lots of researches have been conducted to explore their structural and surface stabilities^26^,^27^,^28^,^29^,^30^, and efforts have been made to study the formation of graphene-like ZnO membranes^31^ and the nucleation behavior of ZnO nanomaterials^32^. On the other hand, coalescence of nanocrystals is an important phenomenon in the early formation stage of nanomaterials. However, direct observation of ZnO NW growth process and the dynamic evolution of the coalescence process of ZnO nanocrystals at atomic scale still lack experimental demonstration.

Here, in order to understand the VS mechanism, the noncatalytic growth of ZnO NWs is studied at atomic scale through *in situ* high resolution TEM (HRTEM) using a FEI Titan 80–300 TEM equipped with a spherical aberration corrector for the objective lens. The atomic resolutional layer-by-layer growth of ZnO nanopillar along [0001] direction is observed under electron-beam (e-beam) irradiation, while no growth is observed along the radial directions, indicating an anisotropic growth mechanism. The coalescence of ZnO nanocrystals is also dynamically observed. Our *in-situ* observations may contribute to the intuitive understanding of the VS mechanism for the catalyst-free NWs growth and coalescence in the nucleation stage of nanocrystals.

## Results

### Layer-by-layer growth

**Figure 1:**
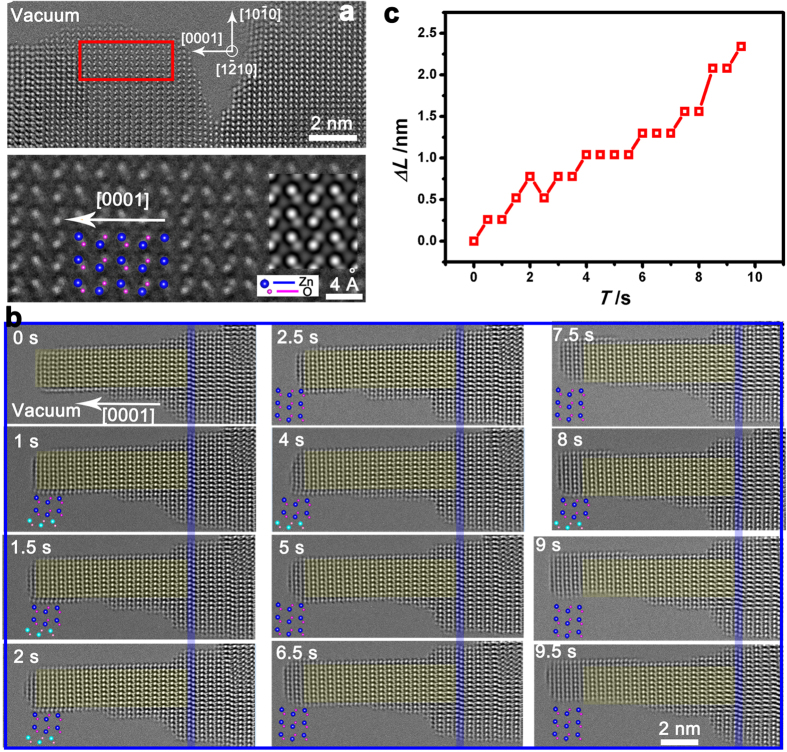
Layer-by-layer growth process for a ZnO nanopillar. (**a**) The HRTEM image (upper) and the magnified image in the red framed region (bottom); the corresponding atomic simulation of this ZnO sample and the atomic model are also superimposed, showing the polarity of this sample toward the vacuum is [0001]. (**b**) Layer-by-layer growth of a ZnO nanopillar under e-beam irradiation; a stacking fault is marked with blue color and the inset schematic atomic models represent the atoms in the outmost layer and inner layers, indicating the back-and-forth movement of the atoms along the outermost 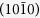 surface; (**c**) The relationship between the length change (*ΔL*) and the e-beam irradiation time (*T*).

The e-beam with an acceleration voltage of 300 kV is used to irradiate the ZnO TEM sample. During our observations, the beam current is 10 nA and the magnification is fixed in all the experiments. Based on the fact that the ZnO film is grown by the pulsed laser deposition (PLD) method and the HRTEM image of the ZnO sample shown in Fig. 1a, the polarity of this sample toward the vacuum is determined to be [0001] (see Methods and Supplementary Information 1).

Figure 1b presents an *in situ* time-series of HRTEM images showing the evolution of a ZnO nanopillar under the e-beam irradiation. The ZnO sample here is acting as the source for the nanopillar growth. The stacking fault at the root of this nanopillar (marked by blue color in Fig. 1b) is used as a reference for our quantitative analysis on the growth process. With the increase of the irradiation time, a layer-by-layer growth of (0002) surface can be clearly observed and 9 new Zn-O atomic layers are formed within 9.5 s. Only the growth along the polar [0001] direction is observed, while no growth is observed along the radial directions, indicating an anisotropic growth. The relationship between the length change (*ΔL*) of the ZnO nanopillar and the irradiation time (*T*) is shown in Fig. 1c and the length change rate is then estimated to be 0.26 nm/s. To confirm the newly grown layers are ZnO other than Zn, we measured the lattice distances of the newly grown layers and found they are the same as that of the original ZnO film (see Supplementary Fig. S1). Moreover, no severe lattice distortion is observed at the interface between the newly grown layers and the original layers along the 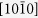 direction, in which the lattice difference between ZnO and Zn is 21%. Therefore, the newly grown layers are indeed ZnO instead of Zn.

Furthermore, as indicated by the insets in Fig. 1b, the surface structure on the bottom outermost 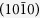 surface are observed to move back-and-forth during the NW growth process, which may correspond to a reversible reconstruction from WZ to the body-centered-tetragonal (BCT) structures on ZnO 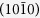 surfaces (see Supplementary Information 3)^28^. Unlike the previous studies in environmental TEM^19^,^20^, no gas is introduced into the chamber and the vacuum at the sample chamber is around 10^−5^ Pa. The source material can only come from the e-beam irradiated ZnO sample. In the VS growth of NWs, atoms and/or molecules can arrive from the vapor to the top of a growing NW in three ways: (1) atoms and/or molecules arrive directly on the top of the growing NW; (2) atoms and/or molecules impinge on the sidewalls of the growing NW and diffuse to the top; (3) atoms and/or molecules impinge on the substrate and diffuse to the NW and then diffuse up along the sidewall to the top. The mass transport process, especially the atoms diffusion process plays an important role in the VS mechanism. The above observed reversible surface reconstruction might be helpful to realize this mass transport process along the nanopillar.

### Surface diffusion process

**Figure 2:**
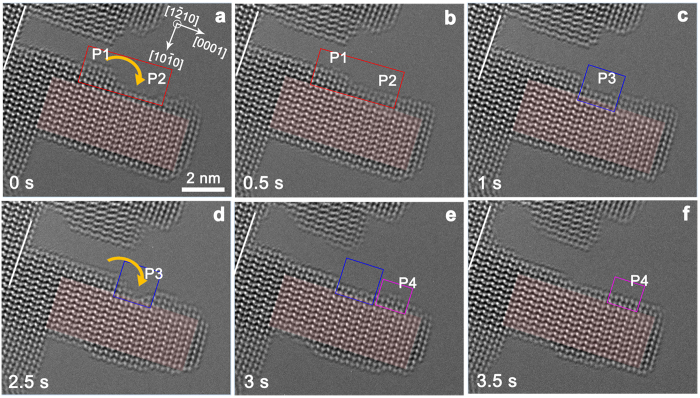
The e-beam induced damage and the diffusion of the surface atoms. (**a**,**b**) The damaged position P2 is filled up by the atoms in position P1 through atom movement; (**c**,**d**) The atoms in position P3 are sputtered away; (**e**) The damaged P3 is filled up by side surface atom migration; (**f**) The atoms in position P4 are sputtered away.

To understand how the source atoms transport to the (0002) surface, other ZnO nanopillars are investigated. As can be seen in Fig. 2, the side 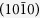 surfaces of the ZnO nanopillars are more sensitive to the e-beam irradiation and the side surface atoms of this nanopillar can be easily sputtered away from their original positions and move along the side surface. The relative contrast of ZnO atoms inside of the nanopillar is stable, which means the thickness of the nanopillar along the e-beam direction doesn’t change. Particularly, as indicated by the arrows in Fig. 2a,b, in order to reduce the total surface energy, the atoms in position P1 can move along the 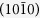 surface to fill up the damaged position P2. Moreover, as shown in Fig. 2c, atoms in position P3 are sputtered away, forming a damaged area (Fig. 2d), which is then filled up by the side atom movement (Fig. 2e). Such analogous sputtering behavior can also be seen in Fig. 2f, where atoms in position P4 are desorbed from the side surface. Since the cleavage energy for the polar surfaces is roughly two times larger than that for the nonpolar surfaces^27^, polar surfaces are more resistant to the e-beam irradiation damage than their nonpolar counterparts. And the sputtered atoms from the nonpolar surfaces can be easily absorbed on the polar surface [0001], leading to the sputtering phenomenon and diffusion of the sputtered adatoms on 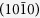 surfaces. These sputtered atoms, which may remain absorbed on the nanopillar surface or be reabsorbed from the vicinity vacuum, and their subsequent migration serve as one of the main sources of the ZnO nanopillar growth.

### Intralayer growth process

**Figure 3:**
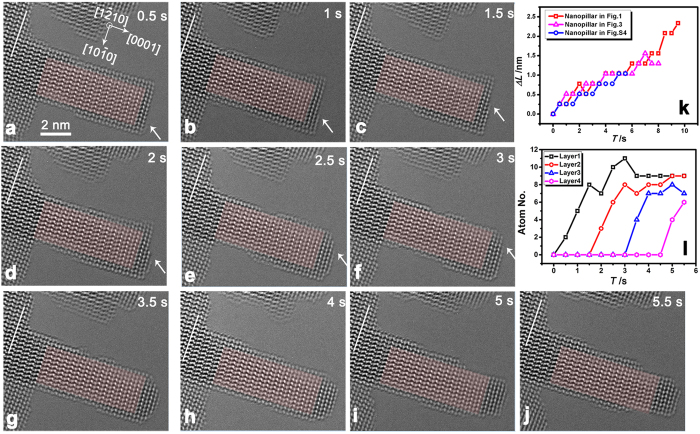
Assemble process of atoms on each growing layer. (**a–j**) The e-beam induced ZnO nanopillar growth. The white arrows indicate the diffusion of atoms on the (0002) surface. (**k**) Comparison of the length change rate of three nanopillars. (**l**) The change of the Zn atom numbers on each growing layer with irradiation the time.

Further irradiation of this ZnO nanopillar leads to the reproducible phenomenon of layer-by-layer growth of the nanopillar along the polar [0001] direction (Fig. 3). Figure 3a–j shows the detailed layer-by-layer growth process and the comparison of the length change rate of this nanopillar with that of the nanopillars shown in Fig. 1 and in Supplementary Fig. S3, demonstrating that the e-beam induced length change rate of these nanopillars are almost the same (Fig. 3k). Importantly, the growth of the top layer on the (0002) surface can be clearly observed (pointed by the white arrow in Fig. 3a–f). Initially, only a small number of atoms attach to the top surface and nucleate a new layer. Then more atoms attach to the edge of the new layer so that the layer grows larger. Another layer can nucleate when the top layer growth has not finished. The top couple of layers grow together until meet the diameter of the nanopillar. The change of Zn atom numbers on each newly grown layer with irradiation time is presented in Fig. 3l, which shows that the growth rate of each layer shares almost the same value. Meanwhile, the e-beam damage to the side 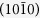 surfaces of the nanopillar and the nearby substrate is also presented, which suggests that the main source material of the layer-by-layer growth comes from the e-beam induced damage of the ZnO sample. The growth process of another ZnO nanopillar is presented in Supplementary Fig. S3, showing similar phenomena. With direct *in situ* HRTEM observations, we find that during the VS growth process, the atoms remaining absorbed and/or being reabsorbed from the vicinity vacuum can reach the top (0001) surface of the nanopillar via a diffusion process along the sidewalls of the nanopillar, and the atoms attaching to the edge of the new (0001) layer cause the intralayer growth.

### Nanocrystal coalescence process

**Figure 4:**
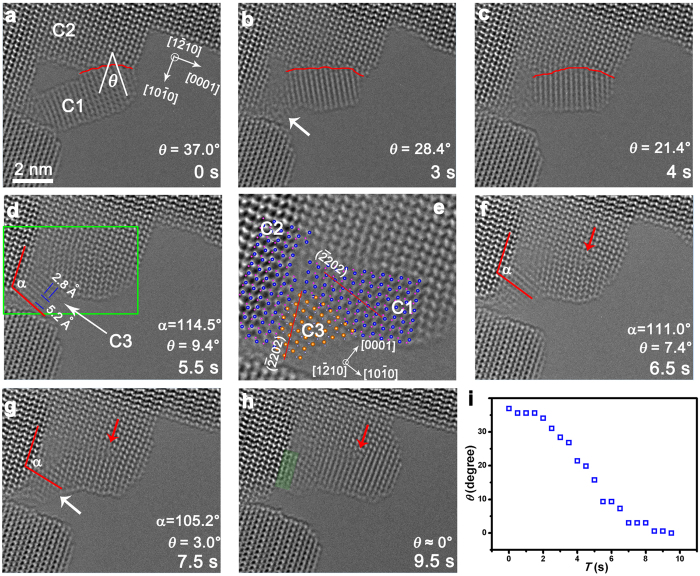
Coalescence of two ZnO crystals. (**a**) HRTEM image showing the angle *θ* between the (0002) planes of C1 and C2. (**b–d**) The rotation of C1 resulting in the decrease of *θ*. The white arrows point the formation process of C3. (**e**) The magnified HRTEM image of the framed area in (**d**) and the corresponding atomic models are superimposed. The 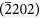 planes of C1 and C3 are denoted by the red lines. (**f–g**) The rotation of C3. (**h**) The atom arrangement of C3. (**i**) The relationship between *θ* and the irradiation time.

Apart from the growth process of one single crystal, nanocrystal coalescence is another crucial process in material growth. The coalescence of two ZnO nanocrystals is also investigated and nanocrystal rotations driven by e-beam irradiation are observed. Figure 4 shows an *in situ* time series of HRTEM images of two ZnO crystals (C1 and C2) with different orientations. The angle between the (0002) surfaces of the two crystals, *θ*, is 37.0° at the beginning. Under e-beam illumination, the smaller ZnO nanocrystal C1 rotates and the crystal boundary moves simultaneously (marked by the red line in Fig. 4a–c) with the reduction of *θ*. An edge dislocation (indicated by a red arrow in Fig. 4f–h) can be clearly observed when *θ* is smaller than 9.4°. Finally, there is almost no angle between these two crystals in Fig. 4h. The change of the angle between these two nanocrystals with irradiation time is shown in Fig. 4i, which demonstrates a much smaller rotation speed at the initial and final stages.

Additionally, probably due to the large strain, the left part of C1 forms a new nanocrystal C3 with size about 2 nm, as indicated by the white arrow in Fig. 4d. By measuring the interplanar spacings, the crystal direction of C3 can be determined (Fig. 4d,e). The magnified image of C1, C2 and C3 in the framed area in Fig. 4d and their corresponding atomic models are shown in Fig. 4e, clarifying that the orientation relationships between C1, C2 and C3 are roughly 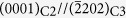 and 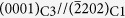, thus the interface energy is minimized (see Supplementary Fig. S4). Along with this recrystallization process, the open space between C1 and C2 gradually disappears. Then, the angle α between (0001)_C2_ and (0001)_C3_ decreased from 114.5° to 105.2°, indicating the rotation of crystal C3 (Fig. 4d–g). Finally, with slight atom rearrangement, the left two layers of C3 are almost in the same direction with C2 (present with the green shadow in Fig. 4h). The interface energy between C3, C2 and C1 in Fig. 4h should be much smaller than that of the large angle grain boundary between C1 and C2 in Fig. 4a. Thus, driven by the reduction of the surface and interfacial energies, a coalescence process is realized through nanocrystal rotation and recrystallization with the help of e-beam exposure.

## Discussion

Now we concern how the above layer-by-layer growth of ZnO nanopillar occurs in the present *in situ* experiments. The source material for nanopillar growth comes from the radiation damage of the ZnO sample. Electron beam induced damage mainly includes (1) atomic displacement and e-beam induced sputtering through knock-on mechanism, (2) electrostatic charging and radiolysis and (3) e-beam heating. The e-beam heating can be ignored here due to a rather low beam current of 10 nA being employed and good thermal conducting condition in our experiments.

For the e-beam induced atomic displacement, if a relativistic electron with energy *E* directly hit on a nucleus, it will transfer a maximum energy *E*_*m*_, given by^33^:


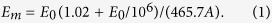


Where *E*_*0*_ is the incident electron energy in eV, *A* is the mass number of the target material. If this maximum energy *E*_*m*_ exceeds the displacement energy *E*_*d*_ of some atoms, the atoms can be displaced to other positions. For the surface atoms, which are easier to be displaced than the atoms inside the materials, if *E*_*m*_ exceeds the threshold energy *E*_*s*_ of some surface atoms, e-beam induced sputtering can occur and the surface atoms are free to leave the specimen or migrate on the surface^30^,^33^. Generally, light and medium-*Z* atoms appear to be vulnerable to sputtering by high energy electrons^33^.

In the present experiment, high energy electron with energy of 300 keV can transfer the maximum energies of 13.0 eV and 53.1 eV to Zn atoms and O atoms, respectively, according to equation (1). For ZnO, the displacement energy *E*_*d*_ for Zn atom and O atom are reported to be 18.5 eV and 41.4 eV, respectively^30^. The surface displacement energy *E*_*s*_ should be much lower than these values. Therefore, the transferred energies are high enough to knock the surface atoms away from their original positions in the specimen, leading to the damage of the side 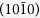 surfaces (Fig. 2). Due to the much larger cleavage energy of polar surfaces^27^, the (0001) surfaces are more resistant to the e-beam irradiation damage. The knocked atoms can be still absorbed on the surface or go into the vacuum and be reabsorbed on the sample surface. The absorbed and reabsorbed atoms can easily diffuse after obtaining enough energy from the incident electrons, leading to the atomic movements shown in Figs 2 and 3. Since the estimated diffusion length of Zn atoms on ZnO sidewalls is over 100 nm^34^, the knocked atoms can migrate on the surface and act as one of the main sources of the growth. These sputtered atoms can be easily absorbed on the polar surface (0001), which possesses a large surface free energy^35^,^36^, resulting in the layer-by-layer growth of ZnO nanopillars along the polar [0001] direction.

Besides, under the exposure of the electrons with rather high energy of 300 keV, the thin semiconducting ZnO sample can be positively charged and the radiolysis reactions can also happen, leading to the desorption of the surface Zn/O atoms^33^,^37^,^38^. Due to the positively charged ZnO surface, these electron-stimulated desorption of O^+^ and Zn^+^, which may be produced by the Knotek-Feibelman (K-F) mechanism, can migrate and diffuse along the surface or into the vacuum.

Therefore, apart from the knocked atoms remaining absorbed on the nanopillar surface, the atoms got into the vacuum by knock-on or by the electrostatic charging and radiolysis processes can be reabsorbed onto the sidewall of the ZnO nanopillar. These reabsorbed atoms can contribute to the growth through the subsequent diffusion process we have discussed above. Considering the relatively large mean free path and the various directions of the sputtered/desorbed atoms, the probability of their direct attachment onto the top (0001) surface from the vacuum should be relatively low compared with the reabsorption onto the side surface and diffusion to the growing tip.

Furthermore, the e-beam induced deposition of amorphous carbon is not likely to form in our experiment due to the rather high energy e-beam, the spread e-beam and the absence of intentionally introduced hydrocarbon molecules^28^,^29^,^39^,^40^. Although carbon can convert ZnO into Zn vapor and CO at a temperature around 950 °C^41^, this high temperature cannot be reached by beam heating effect in our experiment.

From another perspective, our observed results can also help to understand the atom assemble behaviors in the homoepitaxy process (where the substrate and film are of the same chemical species). Thermodynamic considerations, based on the balancing of the free energies of the film surface, substrate surface, and the substrate/film interface, predict a layer-by-layer, Frank-van der Merve, growth mode in the homoepitaxy process^42^. When scrutinizing the atom corporation mode on each layer in Fig. 1 and Supplementary Fig. S3, we find the observed layer-by-layer growth is similar to the epitaxial Frank-van der Merwe growth process. Since growth occurs at the conditions which are often far from equilibrium, and kinetic limitations associated with the finite rates of mass transport processes can greatly affect the actual growth mode, the epitaxy manner of nanopillar in Fig. 2 shows a multilayer growth mode.

## Conclusions

In conclusion, through *in situ* HRTEM, we investigate the growth process of ZnO nanopillars and the coalescence of ZnO nanocrystals at atomic scale. The ZnO nanopillars are observed to grow layer-by-layer along [0001] direction through an anisotropic growth mechanism in the absent of any catalyst. The sputtered surface atoms through knock-on mechanism and electron-stimulated desorption of atoms probably through the K-F mechanism provide the source material for the nanopillar growth. The atoms remaining on the surface or being reabsorbed onto the surface from the vacuum subsequently diffuse along the side 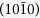 surfaces to the top (0002) surface of the ZnO nanopillar and contribute to the anisotropic layer-by-layer growth. Additionally, a coalescence of ZnO nanocrystals is realized through nanocrystal rotation and recrystallization under e-beam exposure. The present results provide crucial information on the NW growth at atomic level and are helpful for understanding VS mechanism. The dynamic information obtained from the *in situ* TEM observations is expected to broaden our current knowledge regarding the catalyst-free growth mechanism of NWs.

## Methods

### Material growth

High quality ZnO films were prepared on *c*-face sapphire substrates using PLD technique. The high purity ZnO dense target was synthesized via standard ceramic synthesis method. Prior to the deposition process, the background vacuum (around 2 × 10^−6^ Torr) was maintained in the chamber. The substrate temperature was kept at the optimized temperature of 600 °C. During the film growth, the oxygen partial pressure of 10 mTorr was used. A KrF excimer laser (λ = 248 nm) was applied for the ablation, with the fluence of 1.5 J/cm^2^ and repetition rate of 10 Hz. The films were grown to the thickness of ~200 nm for 15 min. The as-grown films were *in-situ* annealed for 10 min in the oxygen atmosphere with the oxygen partial pressure around 200 Torr to decrease the oxygen vacancies and keep the stoichiometric ratio in ZnO films, then followed by cooling down to the room temperature at the rate of 15 °C/min.

The sample quality was characterized by X-ray diffraction (XRD), using a Rigaku Smartlab X-ray diffractometer. The *θ* − *2θ* scanning results for the ZnO film deposited on an Al_2_O_3_ substrate were demonstrated in Supplementary Fig. S5.

### Sample fabrication

The cross-sectional TEM samples were made by traditional method, including cutting, polishing, dimpling and ion milling processes (see Supplementary Fig. S6). Wire saw was used for cutting in order to minimize the sample damage. In the last process of the TEM sample fabrication, ionized Ar gas flow was used to bombard samples for the sake of creating observation window. Initially uniform ZnO film was scratched by high energy ionized Ar gas and some ZnO nanopillars were thus created around the observation window (see Supplementary Fig. S6).

## Additional Information

**How to cite this article**: Li, X. *et al*. Direct Observation of the Layer-by-Layer Growth of ZnO Nanopillar by *In situ* High Resolution Transmission Electron Microscopy. *Sci. Rep.* **7**, 40911; doi: 10.1038/srep40911 (2017).

**Publisher's note:** Springer Nature remains neutral with regard to jurisdictional claims in published maps and institutional affiliations.

## Supplementary Material

Supplementary Information
