# Supplementary material for: Direct Observation of the Layer-by-Layer Growth of ZnO Nanopillar by In situ High Resolution Transmission Electron Microscopy
Source: Sci Rep. 2017 Jan 18;7:40911. doi: 10.1038/srep40911 (PMC5241657; doi:10.1038/srep40911)
Supplement: Supplementary Information [file srep40911-s1.pdf]

# Supplementary Information

## Direct Observation of the Layer-by-Layer Growth of ZnO Nanopillar by *In situ* High Resolution Transmission Electron Microscopy

**Xing Li,[1+] Shaobo Cheng,[2,3 +] Shiqing Deng,[2] Xianlong Wei,[1] Jing Zhu,[2,3]* Qing Chen[1]***

[1]Key Laboratory for the Physics and Chemistry of Nanodevices and Department of Electronics, Peking University, Beijing 100871, P. R. China

[2]National Center for Electron Microscopy in Beijing, School of Materials Science and Engineering, The State Key Laboratory of New Ceramics and Fine Processing, Key Laboratory of Advanced Materials (MOE), Tsinghua University, Beijing 100084, P. R. China

[3]Center for Nano and Micro Mechanics, Tsinghua University, Beijing 100084, P. R. China

*Corresponding Authors: qingchen@pku.edu.cn; jzhu@mail.tsinghua.edu.cn

[+]These authors contributed equally to this work

### 1. Polarity determination of the ZnO nanopillar

HRTEM images were taken by negative spherical aberration correction technique (NCSI) at 300kV accelerating voltage.[1, 2] Based on the experimental parameters applied for acquiring HRTEM image (Fig. 1a), we conduct image simulations by using the multi-slice method[3] with a commercial MacTempas program. During the experiments, the spherical aberration (Cs) was set to about -13 μm, which meets the negative Cs imaging (NCSI) condition.[1] The two-fold astigmatism (A1), three-fold astigmatism (A2) and axial coma (B2) are set to be 0.08 nm, 19.2 nm and 11.3 nm, respectively. The simulation parameters, including aberration parameters listed above, the convergence angle (0.20 mrad), the mechanical vibration (0.04 nm), the ranges of over-focus and the specimen thickness, as well as the sample tilt conditions (slightly tilted around exact $[1\bar{2}10]$ zone axis), are imported as the initial input. The simulated images for a range of specimen thickness (from 10 Å to 50 Å, with a step of 2 Å) and over-focus (from 40 Å to 80 Å, with a step of 2 Å) are shown as output results. Then,

the cross correlation coefficients (CCC), which measure similarity in the pattern between the experimental image and the computed image, are calculated. Since the images to be compared are set to a mean level of 0 and normalized, any scaling of the type $I_{Exp} = a \times I_{Calc} + b$ (where $I_{Expe}$ is the intensity of the experimental image; $I_{Calc}$ is the intensity of each simulated image; a and b are two constants) would give a cross correlation coefficient of 1. Exact fit is given as CCC = 1 and a reverse contrast gives CCC = -1. Through iteratively repeating this process, each parameter can be optimized and the best-fitted simulation image can be found, as shown in the inset in Fig. 1a. Thus, the parameters for the best-fitted simulation image can be determined: the specimen thickness is around 22 Å, the over-focus is optimized as about 54 Å, and the specimen is slightly tilted along [0001] direction, the angle of which is around 7 mrad off the exact $[1\bar{2}10]$ zone axis.

## 2. Determination of ZnO

As shown in Supplementary Fig. S1b, we measure the atomic distance of the inner ZnO and use it as reference to obtain the atomic distance of the newly grown layers. The interlayer distances of the newly grown layers are measured to be 2.8 Å and 5.2 Å, corresponding well with that of ZnO (shown in Supplementary Fig. S1b, c). For eight atoms marked by the colored lines in Supplementary Fig. S1b, c, the lattice difference along the $[10\bar{1}0]$ direction between Zn and ZnO is 4 Å, which is large enough to be distinguished. Besides, there should exists a 21% lattice difference along the $[10\bar{1}0]$ direction between Zn and ZnO (2.31 Å and 2.81 Å) , while no severe lattice distortion is observed at the interface between the original ZnO film and the newly grown layers. These facts indicate that the newly grown layers are ZnO rather than Zn.

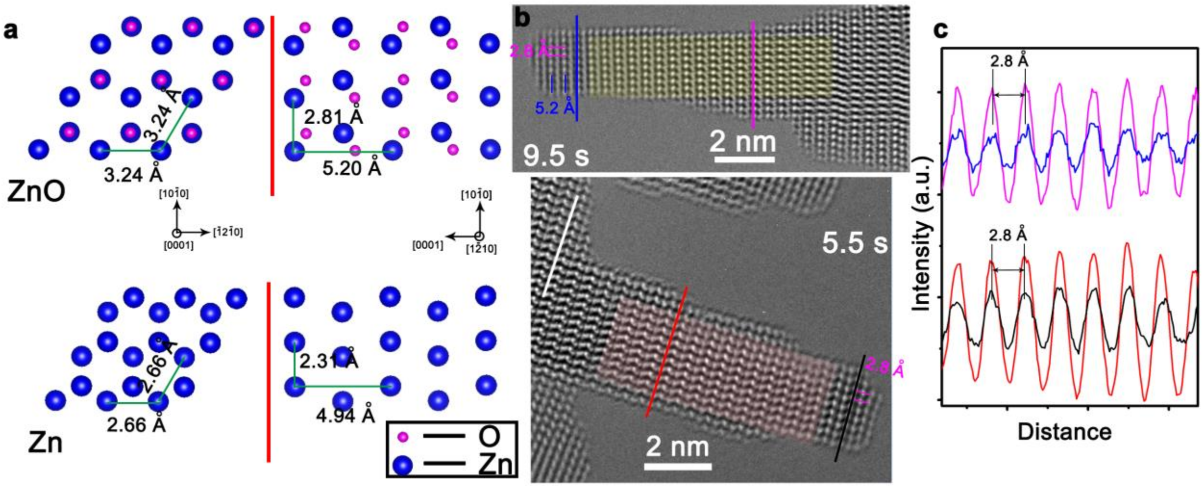


**Supplementary Figure S1. Determining the newly grown layers are ZnO.** (**a**) Atomic models of ZnO and Zn, with their lattice parameters also indicated. (**b**) The measured interlayer distances of the newly grown material, indicating the material is ZnO rather than Zn. (**c**) The intensity profiles for the region marked by the colored lines in (b).

## 3. Reversible Wurtzite-Tetragonal Reconstruction in the ZnO nanopillar

**Supplementary Table S1.** Structure parameters of WZ and BCT phases ZnO [4]

| | Space Group | Lattice constants | Angles | Wyckoff Positions |
|---|---|---|---|---|
| WZ | $P6_3mc$ | a=b=3.25Å, c=5.20 Å | α=β=90°, γ=120° | Zn(0.333,0.667,0), O(0.333,0.667,0.382) |
| BCT | $P4_2/mnm$ | a=b=5.58 Å, c=3.24 Å | α=β=γ=90° | Zn(0.333,0.667,0), O(0.167,0.833,0.5) |

According to the lattice parameters for wurzite (WZ) ZnO and body-centered-tetragonal (BCT) ZnO shown in Supplementary Table S1, atomic configurations of the ZnO $(10\bar{1}0)$

surface before (see Supplementary Fig. S2a) and after (see Supplementary Fig. S2b) WZ-BCT reconstructions in the outmost layers are constructed. The WZ-BCT reconstruction corresponds well with our experimental results (see Supplementary Fig. S2c). During the layer-by-layer growth process, this outmost layer presents reversible WZ-BCT reconstruction behavior

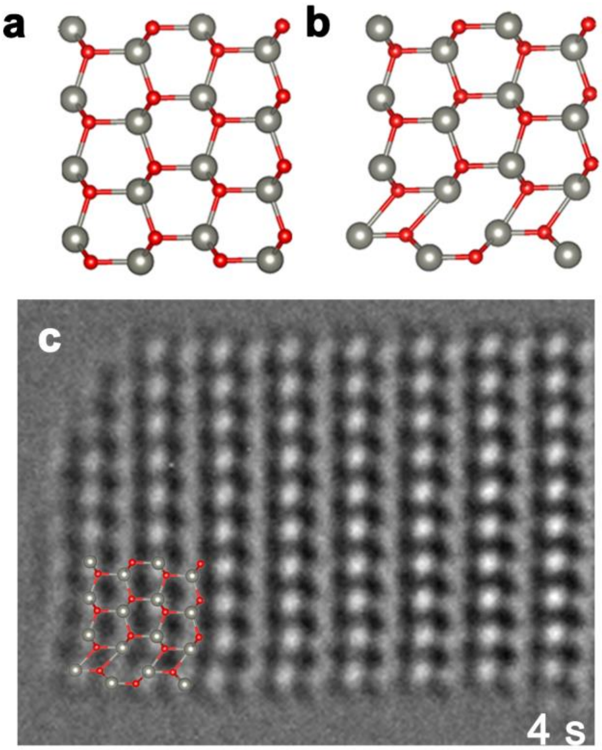


**Supplementary Figure S2. Side surface reconstruction of the ZnO nanopillar.** Atomic models of ZnO $(10\bar{1}0)$ surface before (**a**) and after (**b**) WZ-BCT reconstruction. WZ-BCT reconstruction can be found at the outmost layers as shown in the bottom part of (**b**) and (**c**). (**c**) Magnified image of the top part of the nanopillar after 4s growth in Fig. 1b. Atomic distortions happen because of the unequivalent lattice parameters of WZ and BCT phases ZnO.

Once the surface reconstructions happen, as shown in Supplementary Fig. S2b, the

orientation relationship between WZ and BCT phases is $[0001]_{WZ}$ // $[100]_{BCT}$ and $[2110]_{WZ}$ // $[001]_{BCT}$. Thus, BCT phase ZnO suffers from 7.3% in-plane compressive strain and surface lattice distortion can be expected.

**4. Nanopillar Growth**

Supplementary Fig. S3 shows the layer-by-layer growth process of another ZnO nanopillar. Five Zn-O layers are formed within 8s. Comparing the length change rate with the nanopillars shown in Fig. 1 and Fig. 3 indicates they share almost the same value of 0.26 nm/s.

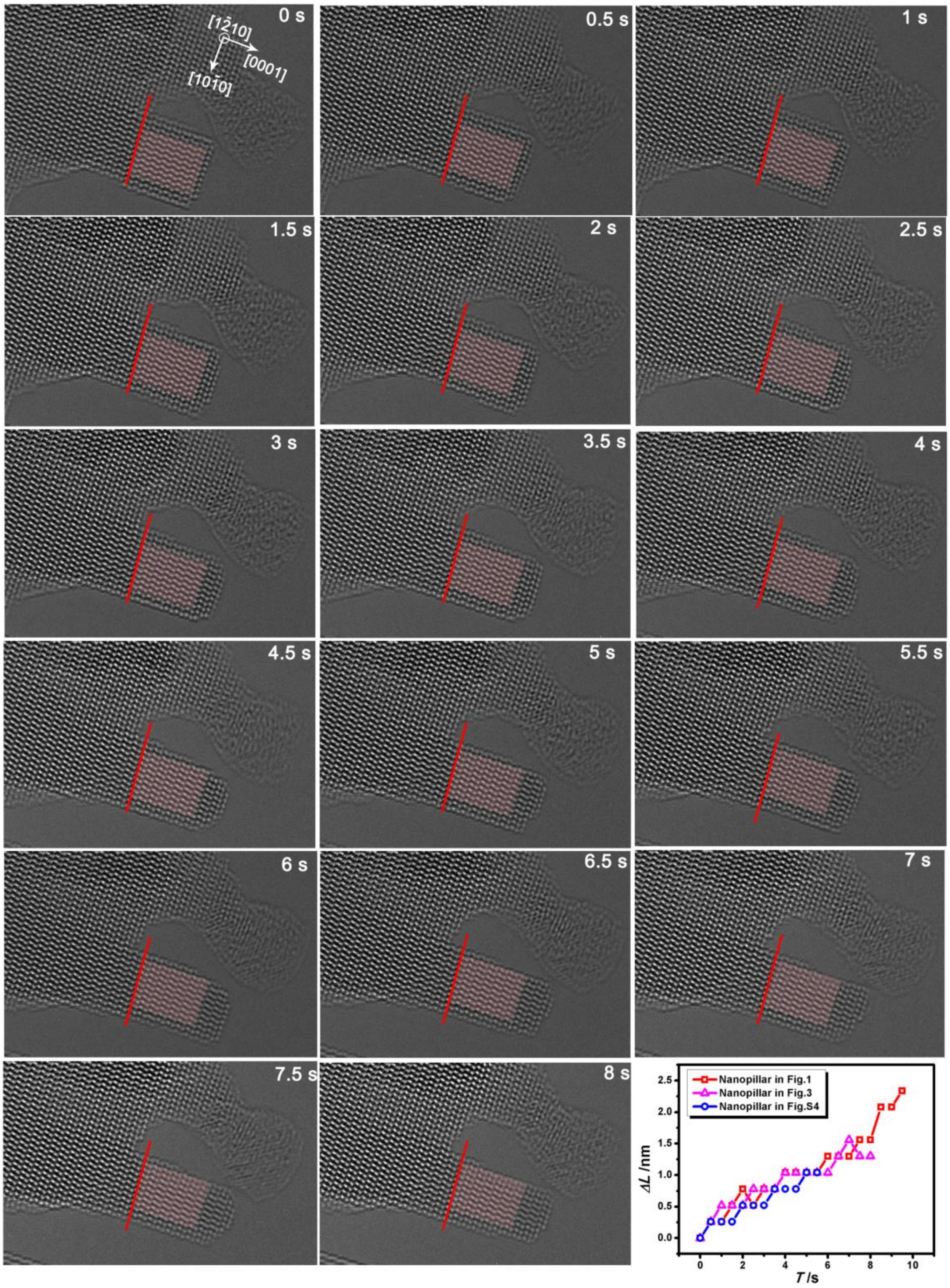


**Supplementary Figure S3. Layer-by-layer growth of a ZnO nanopillar.** The layer-by-layer growth process of another ZnO nanopillar, showing almost the same length change rate with the nanopillars shown in Fig. 1 and Fig. 3.

## 5. Atomic arrangement of (0001) plane and $(\bar{2}202)$ plane of ZnO

By measuring the interplanar spacings, the crystal direction of C3 is determined and the crystal orientation relationships with C1 and C2 are shown in Fig. 4e. According to our results, the orientation relationships between C1, C2 and C3 are $(0001)_{C2}$ // $(\bar{2}202)_{C3}$ and $(0001)_{C3}$ // $(\bar{2}202)_{C1}$. The atomic arrangement of (0001) plane and the $(\bar{2}202)$ plane and their relationship are shown in Supplementary Fig. S4.

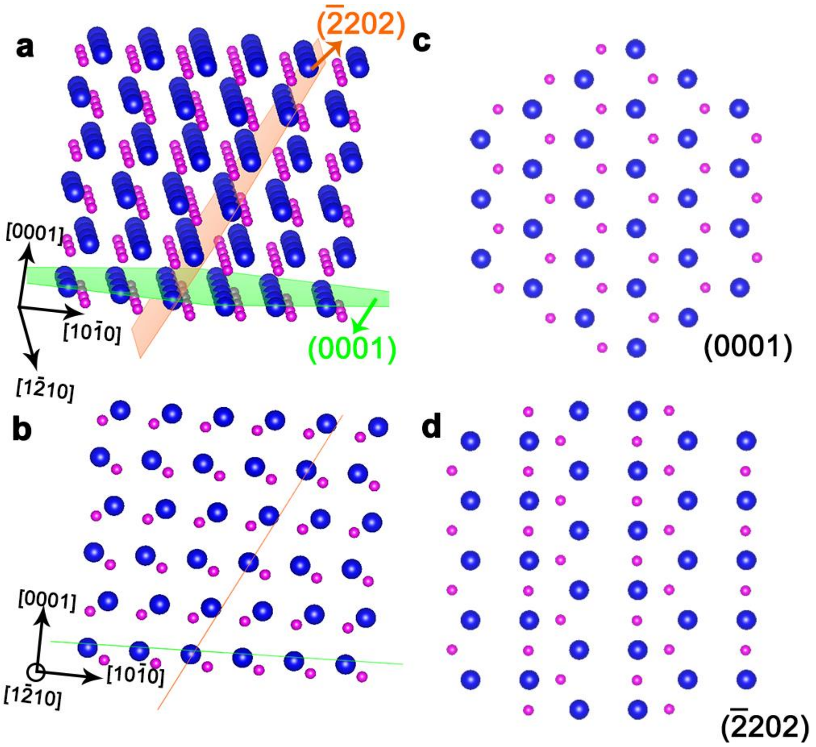


**Supplementary Figure S4. Atomic model of WZ ZnO, showing the atomic arrangement of the (0001) and $(\bar{2}202)$ planes.** (**a**) 3D view of the (0001) and $(\bar{2}202)$ planes. (**b**) Side view of the (0001) and $(\bar{2}202)$ planes. (**c**) The atomic arrangement of (0001) plane. (**d**) The atomic arrangement of $(\bar{2}202)$ plane.

## 6. XRD Characterization of the ZnO sample

The sample quality was characterized by X-ray diffraction (XRD), using a Rigaku Smartlab X-ray diffractometer. The *θ-2θ* scanning result for the ZnO film deposited on $Al_2O_3$

substrate is demonstrated in Supplementary Fig. S5. Only (000n) peaks can be seen, implying the film was grown with the out-plane direction parallel to that of the substrate. To verify the in-plane symmetry of the film, ZnO $(11\bar{2}2)$ plane and $Al_2O_3$ $(11\bar{2}3)$ plane, which share the same in-plane projection component, were selected for the *Φ*-scanning experiments. The peaks for both ZnO and $Al_2O_3$ peaks are separated by 60°, showing six-fold rotational symmetry of (0001) plane. The ZnO peaks are 30° off-set to $Al_2O_3$ peaks, indicating a 30° in-plane rotation of the film with respect to the substrate. Thus, the epitaxial relationship of the ZnO film on the $Al_2O_3$ substrate can be characterized to be ZnO [0001] // $Al_2O_3$ [0001] and ZnO $[10\bar{1}0]$ // $Al_2O_3[11\bar{2}0]$.

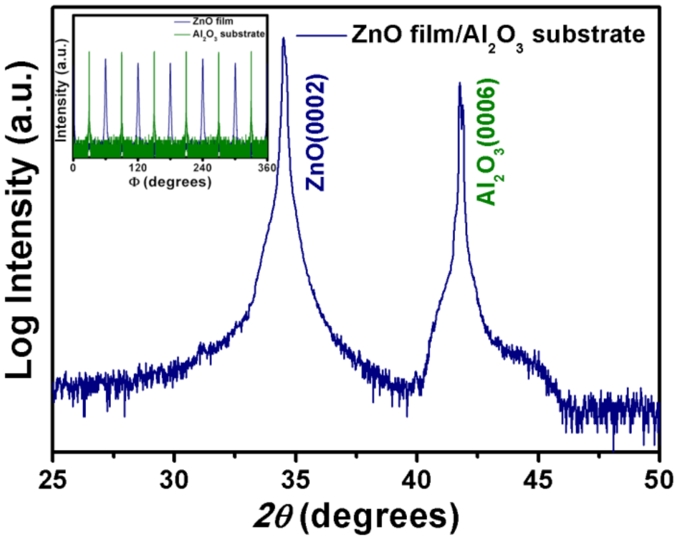


**Supplementary Figure S5. The XRD spectra of a ZnO film/$Al_2O_3$ substrate sample.** Only (000n) peaks, both for the $Al_2O_3$ substrate and the ZnO film, can be detected in *θ-2θ* scanning. The *Φ*-scans results are demonstrated in the inset, implying a 30° in-plane rotation of the ZnO film on the $Al_2O_3$ substrate.

## 7. TEM Specimen Fabrication Process

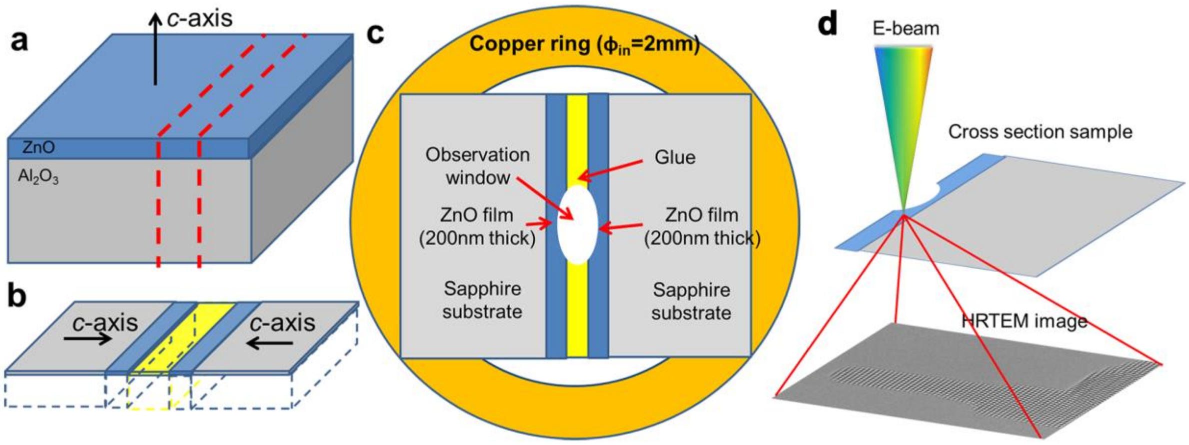


**Supplementary Figure S6. TEM sample fabrication process.** (**a**) A wire saw was used to cut the thin film sample. The cutting positions are indicated by the red dashed lines. (**b**) The cutted parts are rotated by 90 ° and bounded by glue (shown in yellow). The sample is thinned by abrasive paper. (**c**) The thinned sample is mounted to a copper ring. In the middle part of the sample, an observation window is opened by ion milling process. (**d**) Schematic diagram showing the experimental geometry.

## References

1. Urban, K. W. Studying Atomic Structures by Aberration-Corrected Transmission Electron Microscopy. *Science* **321**, 506-510 (2008).

2. Cheng, S., Li, M., Meng, Q., Duan, W., Zhao, Y. G., Sun, X. F., Zhu, Y. M., Zhu, J. Electronic and Crystal Structure Changes Induced by In-plane Oxygen Vacancies in Multiferroic $YMnO_3$. *Phys. Rev. B* 93, 054409 (2016).

3. Cowley J M. In *Diffraction Physics*. 3rd ed. (Amsterdam: Elsevier, 1995).

4. Wang, J., Kulkarni, A. J., Sarasamak, K., Limpijumnong, S., Ke, F. J., Zhou, M. Molecular Dynamics and Density Functional Studies of a Body-Centered-Tetragonal Polymorph of ZnO. *Phys. Rev. B* **76**, 172103 (2007).
